# COVID-19 hot-spot strategy: a special innovation in pandemic response, Oyo State Nigeria

**DOI:** 10.1186/s12889-022-12675-2

**Published:** 2022-02-04

**Authors:** Enya Bassey Bassey, Walter Kazadi Mulomb, Ahmed Mohamed Ahmed Khedr, Rex Gadama Mpazanje, Rosemary Ifeoma Onyibe, Olufunmilola Olawumi Kolude, Oluwadare Marcus, Oluwabukola Alawale, Omotunde Ogunlaja, Adeoluwa Iyanda Oluwatobi, Ayodeji Tella Adedamola, Suliat Olanike Olayiwola, Taiwo Olabode Ladipo

**Affiliations:** 1 World Health Organization (WHO) Nigeria Country Office, UN House, Plot 617/618, Diplomatic Drive, Central Business District, PMB 2861, Garki, Abuja, Nigeria; 2 Oyo State COVID-19 Emergency Operation Center, Ministry of Health, Yemetu, Ibadan, Nigeria

**Keywords:** Hotspot strategy initiative, COVID-19, Surveillance

## Abstract

**Background:**

COVID-19, caused by severe acute respiratory syndrome coronavirus 2 (SARS-CoV-2) has evolved into a pandemic. Oyo state, Nigeria with a population of 9,233,010, recorded the first case of COVID-19 on the 12th of March 2020 and it is among the highest contributing States to the nation’s burden of the disease with 3267 confirmed cases, including 40 deaths as of date, with an overall test positivity rate of 18.1%, far higher compared to the National average within a limited period from recorded index case. A ‘Hotspot strategy’ was designed by the Presidential Task Force on COVID-19 and Oyo State was selected to implement the strategy through upscaling case detection, isolation and treatment, quarantine of contacts and strengthening public health and social measures.

**Methods:**

We used a descriptive cross-sectional survey of 3 identified hotspot Local Government Areas (LGAs) in Oyo State using mobile phones under Surveillance, Outbreak Response Management and Analysis System (SORMAS) platform to collect data from October to December 2020. Interventions comprised of enhanced active case search, contact line listing, contact investigation, and contact follow-up as well as to facilitate data collection and entry, community sensitization and management of alert/rumors. Baseline information and that after the 3-month period was then analyzed with the descriptive statistics presented.

**Results:**

The implementation of the hotspot strategy was shown to have had a major impact in Irepo LGA, where more than a 100% increase in samples tested, confirmed cases, contacts listed and contacts followed were recorded, while there were no significance changes noticed in Ibadan North and Lagelu LGAs. However, test positivity rates among contacts were found to be quite high in Ibadan North LGA (48%), compared to the other two, even though Lagelu LGA (5.7%) tested more contacts than Ibadan North.

**Conclusion:**

The observed increase in number of samples tested, cases confirmed, contact listed and investigated as well as test positivity rate in the 3 LGAs after the intervention implies that the hotspot strategy can be said to have contributed positively to the sensitivity of COVID-19 surveillance in Oyo State, Nigeria. This implies that strengthening this ‘hotspot strategy’ may be a key area of focus to improve COVID-19 surveillance sensitivity and response and in turn may help in breaking the transmission and bringing the pandemic to a halt.

## Background

COVID-19 caused by severe acute respiratory syndrome coronavirus 2 (SARS-CoV-2) evolved into a pandemic. On the 27th of February 2020, Nigeria recorded its COVID-19 index case [1]. Following the detection of the index case, the Nigeria Centre for Disease Control (NCDC) and World Health Organization (WHO), responsible for the control of disease outbreaks activated a multi-sectorial National Emergency Operations Centre (EOC) to oversee the country’s response to the pandemic. Subsequently, the Presidential Task Force on the control of COVID-19, initially given a 6-months mandate, was inaugurated on the 17th of March 2020 as the government’s focal point of effort in tackling COVID-19 pandemic [2] and this was followed by activation of EOCs by all states in the country. Hotspot strategy came into play in August 2020, after the 2-day Mid-Term Retreat organized by the presidential taskforce and the strategy form an important aspect of the detailed roadmap of key initiatives and activities for improving COVID-19 response in Nigeria.

In Oyo state, the index case of COVID-19 was recorded on the 12th of March 2020, with 3761 confirmed cases and 46 deaths at the end of epidemiological week 50 being December 13, 2020. This is about 9 months after recording the index case. As at this period, the State ranked 5th among the contributing State to the nation’s burden of the disease and was responsible for 5.1 and 3.8% respectively of all COVID-19 recorded cases and death in Nigeria [3]. The test positivity rate (TPR) of Oyo State as at that week 502,020 was 13.1% and this was comparatively far higher than the National average of 8.5% for the same period. The case fatality rate (CFR) of the State however was 1.2%, a little below the National average of 1.6% for the same period. The State has been using the platform of the EOC to drive response activities which have since commenced to combat the spread of the disease in Oyo State.

Despite these efforts, the COVID-19 surveillance systems in most LGAs in the State was highly suboptimal which could be indicative of undetected ongoing transmission, while other LGAs have become hotspots for transmission of the virus. A major challenge faced in implementing this strategy was the state inadequate healthcare workforce during the outbreak [4]. Some Countries like New Zealand, Australia, Taiwan, Thailand, investigated by Furuse *at al*., (2021) [5] that have conducted testing with great intensity, have been able to maintain low number of COVID-19 cases. It is therefore imperative to strengthen the existing surveillance system by the implementation of the “Hotspot Strategy” put together by WHO in the State to help address existing gaps identified in the system.

The Hotspot strategy is a surveillance initiative conceptualized to identify areas with weak COVID-19 surveillance system, with the purpose of increasing the surveillance sensitivity. The Hotspot strategies involve a beam focus on LGAs where there is high number of cases and community transmission is well entrenched and accelerating in an effort to slow the spread of the virus and protect vulnerable people most at risk through upswing active case search, contact tracing and testing.

This strategy requires the work and effort of all levels of government, working in partnership with NGOs, community networks, volunteers and health workers. It also requires every individual to take responsibility to ensure that they are protecting themselves, and their loved ones and help to slow the spread.

For Oyo State, each hotspot LGA is overseen by a WHO officer, reporting back to the State EOC on plans and progress regularly. Each report focuses on issues including active surveillance, identification of persons of interest, identification of points of entry and screening of travelers, case detection and notification, case investigation, sample collection and testing, case management, identification and institution of isolation and treatment centers, contact tracing and follow-up, risk communication and community engagement, Operationalization of the State Rapid Response Team (RRT), Decentralization of RRTs to LGAs. The strategy included interventions to strengthen surveillance system sensitivity through upswing active case search, contact tracing and testing to slow the spread of the virus and protect vulnerable people most at risk. Here we describe an innovative surveillance strategy implemented in the context of the hot spot strategy and its impact on the community transmission in Oyo State.

The aim of this paper is to evaluate the impact of the hotspot strategy a special innovation in Oyo State at increasing COVID-19 case detection, reporting and investigation in the vulnerable communities.

## Materials and methods

### Study location

This study location is Ibadan North, Irepo and Lagelu LGAs. Two of the LGAs are predominantly urban while one is rural with an estimated population of 953,264 [6].

### Study design

A cross-sectional survey was conducted using mobile phones under Surveillance, Outbreak Response Management and Analysis System (SORMAS) platform from October to December 2020. SORMAS is an open-source mHealth (mobile health) system that organizes and facilitates infectious disease control and outbreak management procedures in addition to disease surveillance and epidemiologic analysis for all administrative levels of a public health system [7, 8]. It provides an out-of-the-box solution for users to build a data collection form or survey, collect the data on a mobile device, send it to a server, aggregate the collected data on a server, analyze the data and extract it in useful formats of Data Collection Tools and Techniques.

### LGA selection criteria

LGAs with high number of active cases ≥100 active cases; LGAs with greater than ≥20% positivity rate with a minimum of 10 new cases in the 2-week period and ≥ 50% increase in new cases of COVID-19 with a minimum of 15 cases at baseline.

### Personnel

WHO provided funding for the recruitment and training of 4 senior field supervisors. In additional three hundred and sixty volunteer community mobilizers, three hundred and fifty (350) Community Informants and two hundred and twenty-eight (228) health workers were engaged and trained to conduct community active case search, Supportive Supervision, daily data collection, Sample collection and entry into the SORMAS Platform and in the management of alert/rumors. They were also engaged in sensitization of community leaders, market women and in the distribution of COVID-19 IEC materials.

### Data collection tools

To effectively facilitate data collection and entry, certain data tools were utilized. Case investigation forms were used to investigate suspected cases prior to sample collection. Contact investigation, contact line listing, and contact follow up form were used for investigation, line listing and follow up of contacts respectively. Rumor logs were used by the DSNOs to record details of rumors and alerts escalated by the community informants. Community active case search forms were used by the VCMs from house to house for COVID-19 active case search while the health facility active case search forms were used in the health facilities for COVID-19 active case search.

### Data analysis

The data were transferred from the server to Excel spreadsheets and thereafter to PASW Statistics 18.0 for cleaning and quality checks. In this paper, we present basic descriptive statistics drawn from the collected data with respect to household with symptoms, age, and sex.

## Results

Table 1 shows the characteristics of COVID-19 cases reported to the surveillance system in the Hotspot LGAs. It compares baseline data and data generated at the end of the special intervention. It summarizes data generated in the following key areas: Samples tested, Confirmed Cases, Contacts line listed, and contacts followed up in the three Hotspot LGAs.

**Table 1:** Distribution of COVID-19 cases reported to the Surveillance System in Hotspots LGAs, 2021

Characteristics	Ibadan North	Irepo	Lagelu
	Baseline/Final (%)*	Baseline/Final (%)*	Baseline/Final (%)*
**Samples Tested**			
Male	1487/1635 (10.0)	6/131 (> 100)	615/686 (11.5)
Female	1328/1477 (11.2)	0/123 (> 100)	744/801 (7.7)
**Confirmed Cases**			
Male	210/225 (7.1)	1/4 (> 100)	116/125 (7.8)
Female	218/231 (6.0)	0/3 (> 100)	139/148 (6.5)
**Contacts Listed**			
Male	72/81 (12.5)	0/16 (> 100)	80/95 (18.8)
Female	91/132 (45.1)	0/9 (> 100)	104/128 (23.1)
**Contacts Followed**			
Male	58/77 (32.8)	0/16 (> 100)	76/91 (19.7)
Female	79/103 (30.4)	0/9 (> 100)	89/101 (13.5)
**Contact to case ratio**			
	0.4/0.5 (21.1)	0/3.6 (> 100)	0.7/0.8 (13.9)

*Source:* Surveillance, Outbreaks Response Management and Analysis (SORMAS), ^*^% Point difference

At baseline, 2815 samples had been tested in Ibadan North LGA. After special intervention, there was 10.6% increase in samples tested with a total of 3112 samples tested. Irepo LGA had greater than 100% increase in samples tested with 6 samples tested at baseline and 254 at the end of the hotspot strategy implementation. Lagelu LGA had 9.6% increase in number of samples tested with 1359 samples tested at baseline and 1487 at the end of the intervention. More males were tested in Lagelu and Irepo LGAs, while more females were tested in Ibadan North LGA.

Comparing baseline data with data generated at the end of the special intervention, Ibadan North LGA had 6.5% increase in confirmed cases with 28 confirmed cases during the period of intervention. More males were affected than females. Irepo LGA showed greater than 100% increase in confirmed cases with 1 case at baseline and 7 cases after intervention. Lagelu LGA had 7.1% increase in confirmed cases with 18 cases during the period of intervention. The sex ratio for confirmed cases in Irepo and Lagelu LGAs during the period of intervention is 1:1.

In Ibadan North LGA, 50 contacts were listed during the period of the strategy implementation, of which 43 were followed up. There was a 30.7% change in number of contacts listed and 31.4% change in contacts followed. In Irepo LGA, no contacts had been listed or follow up prior to special intervention during which a total of 25 contacts were listed with a male to female ratio of 3:2. All listed contacts in Irepo LGA were followed. Lagelu LGA had 39 contacts listed during the period of intervention of which 27 were followed up. There was a 21.2% increase in contacts listed and 16.4 increase in contacts followed. Ibadan North, Irepo and Lagelu LGAs respectively followed up 86, 100 and 69.3% of contacts listed.

The contact to case ratio increased from the baseline value by 21.1, > 100 and 13.9% for Ibadan North, Irepo and Lagelu LGA.

Table 2 shows the Test positivity rate (TPR) among recorded contacts listed and followed by the surveillance system in the Hotspot LGAs.

**Table 2:** COVID-19 Test Positivity Rate (TPR) Among Recorded Contacts in Hotspots LGAs, 2021

Characteristics	Ibadan North	Irepo	Lagelu
Contacts tested	376	25	407
Number Positive	183	0	23
Positivity Rate	48.7%	0%	5.7%

Ibadan North had a positivity rate of 48.7% among contacts listed. None of the contacts in Irepo LGA tested positive. Lagelu LGA had 5.7% positivity rate among contacts listed.

## Discussion

This paper assesses the impact of the Hot Spot Strategy on improving COVID-19 surveillance sensitivity in Oyo State, Nigeria.

A very strong and sensitive surveillance system is important for case detection in high and low burden communities [9]. The status of COVID-19 surveillance in the state necessitated the conceptualization and implementation of a strategy aimed at improving the sensitivity of the surveillance system.

Considering the World Health Organization (WHO) recommendations regarding the need to increase the number of COVID-19 tests [10], there are 2 laboratories for COVID-19 testing in the state, both of which are located in one of the hotspots LGAs (Ibadan North). This mean that sample collection and testing was readily accessible. Prior to the special intervention, there was no sample collection site in Irepo and Lagelu LGAs. This negatively affected case detection in these 2 LGAs, but much more in Irepo LGA which is approximately 270 km away, compared to Lagelu LGA which is 39 km away from the closest testing center. The engagement of community mobilizers, training of Health workers on case detection and VCMs for house-to-house case search, as well as the establishment of sample collection sites in these LGAs brought about over 100% increase in the number of samples collected and tested. In addition, risk communication and community engagement activities such as the sensitization of community leaders and market women, alongside the distribution of COVID-19 IEC materials also increased the awareness of community members and aided their willingness to go for testing. However, the relatively low increase observed in Ibadan North and Lagelu could be attributed to the already existing COVID-19 response activities put in place as they are part of the 9 LGAs prioritized in the state.

During the period of the special intervention, Ibadan North had the highest number of confirmed cases (28) which accounted for a 6.5% increase. This could be as a result of the LGA being the epicenter of the pandemic in the state. However, greater than 100% increase was observed in Irepo LGA with 6 laboratory confirmed cases during the period of the intervention. This increase is linked to the increase number of testing and pointed to the fact that there was likely ongoing transmission of the COVID-19 virus that was previously undetected.

The World Health Organization (WHO) recommended the need to intensify isolation and tracing of contacts [10] in order to reduce transmission and one of the key areas of the hotspot strategy was the engagement of contact tracers and VCMs trained on entry of contact tracing data into the SORMAS database. The contact to case ratio though still a far cry from the expected, increased from the baseline value by 21.1, > 100 and 13.9% for Ibadan North, Irepo and Lagelu LGA. This is an indication that the hotspot strategy has aided in improving contact tracing and isolation.

Considering the percentage of contacts that tested positive to COVID-19 among all contacts that were recorded in these 3 Hotspot LGAs, it was observed that Irepo LGA had no positive cases among the contacts, this could be linked to the low testing of recorded contacts [11] as only 25 recorded contacts were tested. However, even though Lagelu LGA tested more contacts than Ibadan North, the positivity rate in Lagelu was 5.7% while the positivity rate for Ibadan North was 48.7%. The average positivity rate of COVID-19 according to WHO among any demographic group is 5% [12] and a high positivity rate suggests high coronavirus infection rates which points to high transmission in Ibadan North LGA. This can be linked to cosmopolitan mobility, risky behaviors and inter-mixing at social gatherings, which eventually makes Ibadan North LGA vulnerable to transmissions compared to the other 2 LGAs [13]. Another factor could be rapid in-migration which could reduce house shortages and result in higher transmission due to population density [14]. Higher occupant density in built-up environments and increased indoor activity raised the human-to-human contact [15] and may also explain the high positivity rate observed for Ibadan North LGA. Further study may explore using the test positivity rate information to predict case count and epidemic dynamics.

## Conclusion

The observed increase in number of samples tested, cases confirmed, contacts listed and investigated as well as test positivity rate in the 3 LGAs after the intervention, the hotspot strategy can be said to have contributed positively to the sensitivity of COVID-19 surveillance in Oyo State, Nigeria. Strengthening this ‘hotspot strategy’ may therefore be a key area of focus to improve COVID-19 surveillance sensitivity which could be used to predict imminent case count increases. This can be use in strengthening the COVID-19 response and in turn may help in breaking the transmission and bringing the pandemic to a halt.

### Limitations

The study area/scope was limited to only three Local Government Areas (LGAs), accounting for only 9% of the state’s LGAs. The findings and conclusions of a cross-sectional survey conducted in only three LGAs are insufficient to be translated for the entire state. This is further supported by the differences in population demographics, geography, and socioeconomic factors found in the other LGAs. A broader scope/study area would have provided much more substantial evidence for the COVID-19 hotspot strategy’s efficacy.

The research was conducted over a three-month period. A longer survey period, as well as a broader scope, would have provided more evidence of the effectiveness of the hotspot strategy.

## Data Availability

The data were generated as part of the activities supporting disease surveillance and routine immunization in Nigeria. The data are kept at the WHO server and are subject to protection.

## References

[1] CDC. Coronavirus Disease 2019 (COVID-19): How COVID-19 Spreads. Accessed 27 Jan 2020.

[2] Presidential Task Force on COVID-19: Mid Term Report. 2020. final version. Prepared by the PTF Secretariat and Price Waterhouse Coopers.

[3] An update of COVID-19 outbreak in Nigeria for week 50. 2020. https://ncdc.gvo.ng/diseases/sitreps.

[4] Lin X, Rocha ICN, Shen X, Ahmadi A, Lucero-Prisno DE (2021). Challenges and strategies in controlling COVID-19 in mainland China: lessons for future public health emergencies. J Soc Health.

[5] Furuse Y, Ko YK, Ninomiya K, Suzuki M, Oshitani H (2021). Relationship of test positivity rates with COVID-19 epidemic dynamics. Int J Environ Res Public Health.

[6] National Population Commission (NPC) and ICF (2019). Nigeria Demographic and Health Survey 2018.

[7] Tom-Aba D, Silenou BC, Doerrbecker J, Fourie C, Leitner C (2020). The surveillance outbreak response management and analysis system (SORMAS): digital health global goods maturity assessment. JMIR Public Health Surveill.

[8] Tom-Aba D, Toikkanen SE, Glöckner S, Adeoye O, Mall S, Fähnrich C (2018). User evaluation indicates high quality of the surveillance outbreak response management and analysis system (SORMAS) after field deployment in Nigeria in 2015 and 2018. Stud Health Technol Inform.

[9] Tom-Aba D, Nguku PM, Arinze CC, Krause G (2018). Assessing the concepts and designs of 58 mobile apps for the management of the 2014-2015 West Africa Ebola outbreak: systematic review. JMIR Public Health Surveill.

[10] Ibrahim NK (2020). Epidemiologic surveillance for controlling Covid-19 pandemic: Types, challenges and implications. J Infec Public Health.

[11] Martinez-Fierro ML, Rios-Jasso J, Garza-Veloz I, Reyes-Veyna L, Cerda-Luna R, Duque-Jara I (2021). The role of close contacts of COVID-19 patients in the SARS-CoV-2 transmission: an emphasis on the percentage of nonevaluated positivity in Mexico. Am J Infect Control.

[12] Cito F, Amato L, Di Giuseppe A, Danzetta ML, Iannetti S, Petrini A (2020). A COVID-19 hotspot area: activities and epidemiological findings. Microorganisms..

[13] Saker L, Lee K, Cannito B, Gilmore A, Campbell-Lendrum DH. Globalization and infectious diseases: a review of the linkages. London: World Health Organization; Centre on Global Change and Health London School of Hygiene & Tropical Medicine; 2004. No. TDR/STR/SEB/ST/04.2

[14] Lee VJ, Ho M, Kai CW, Aguilera X, Heymann D, Wilder-Smith A (2020). Epidemic preparedness in urban settings: new challenges and opportunities. Lancet Infect Dis.

[15] Andrews JR, Morrow C, Walensky RP, Wood R (2014). Integrating social contact and environmental data in evaluating tuberculosis transmission in a south African township. J Infect Dis.

